# Fully Online Multicommand Brain-Computer Interface with Visual Neurofeedback Using SSVEP Paradigm

**DOI:** 10.1155/2007/94561

**Published:** 2007-07-30

**Authors:** Pablo Martinez, Hovagim Bakardjian, Andrzej Cichocki

**Affiliations:** Laboratory for Advanced Brain Signal Processing, Brain Science Institute RIKEN, Wako-Shi, Saitama 351-0198, Japan

## Abstract

We propose a new multistage procedure for a real-time brain-machine/computer interface (BCI). The developed system allows a BCI user to navigate a small car (or any other object) on the computer screen in real time, in any of the four directions, and to stop it if necessary. Extensive experiments with five young healthy subjects confirmed the high performance of the proposed online BCI system. The modular structure, high speed, and the optimal frequency band characteristics of the BCI platform are features which allow an extension to a substantially higher number of commands in the near future.

## 1. INTRODUCTION

A brain-computer interface (BCI), or a brain-machine interface (BMI), is a system that acquires and analyzes brain signals to create a high-bandwidth communication channel in real time between the human brain and the computer or machine [1–5]. In other words, brain-computer interfaces (BCI) are systems that use brain activity (as reflected by electric, magnetic, or metabolic signals) to control external devices such as computers, switches, wheelchairs, or neuroprosthetic extensions [6–12]. While BCI research hopes to create new communication channels for disabled or elderly persons using their brain signals [1, 2], recent efforts have been focused on developing potential applications in rehabilitation, multimedia communication, and relaxation (such as immersive virtual reality control) [13, 14]. Today, BCI research is an interdisciplinary endeavor involving neuroscience, engineering, signal processing, and clinical rehabilitation, and lies at the intersection of several emerging technologies such as machine learning (ML) and artificial intelligence (AI). BCI is considered as a new frontier in science and technology, especially in multimedia communication [1–18].

The various BCI systems use different methods to extract the user's intentions from her/his brain-electrical activity, for example:

- measuring the brain activity over the primary motor cortex that results from imaginary limbs and tongue movements [3, 5],
- detecting the presence of EEG periodic waveforms, called steady-state visual evoked potentials (SSVEP), elicited by multiple flashing light sources (e.g., LEDs or phase-reversing checkerboards) [6–18]
- identifying characteristic event-related potentials (ERP) in EEG that follow an event noticed by the user (or his/her intention), for example, P300 peak waveforms after a flash of a character the user focused attention on [1–3].

In the first approach, the usually exploited features of the brain signals are their temporal/spatial changes and/or the spectral characteristics of the SMR (sensorimotor rhythm) oscillations, or the mu-rhythm (8–12 Hz), and the beta rhythm (18–25 Hz). These oscillations typically decrease during movement or when preparing for movement (event-related desynchronization, ERD) and increase after movement and in relaxation (event-related synchronization, ERS). ERD and ERS help identify those EEG features associated with the task of motor imagery EEG classification [3, 5].

While the first example (i) relies on imaginary actions to activate the corresponding parts of the motor cortex, the second (ii) and third (iii) examples involve actual selective stimulation in order to increase the information transfer bit rates [3].

Steady-state visual evoked potentials are the elicited exogenous responses of the brain under visual stimulations at specific frequencies. Repetitive stimulation evokes responses of constant amplitude and frequency. Each potential overlaps another so that no individual response can be related to any particular stimulus cycle. It is presumed therefore that the brain has achieved a steady state of excitability [19].

Applications of SSVEP on BCI were proposed by Middendorf [6] and applied later by other groups [7–18, 20]. Previously cited BCI systems, except the approach done by Materka and Byczuk [10], have in common that they are based on spectrum techniques for feature extraction instead of time domain techniques. And all of them use sources of the stimuli (flickering patterns, LED...) in a fixed spatial position.

Comparing to previous SSVEP BCI, our system is based mainly on the temporal domain combining of a blind source separation (BSS) algorithm for artifact rejection and tuned microbatch filtering to estimate the features to be used with a classifier, in our case a fuzzy neural network classifier.

Also, in our design, the sources of stimulus are moving (adding extra complexity), and we have shown that it is possible to perform also a robust BCI for moving flickering targets.

In general, the SSVEP-BCI paradigm has the following potential advantages and perspectives.

1. It offers the possibility of high performance (information rate) with minimal training time and low requirements from the subject.
2. The carefully designed SSVEP-BCI system can be relatively robust in respect to noise and artifacts. Artifacts may cause performance degradation; however they can be removed or reduced using advanced signal processing techniques like BSS. Also, blink movement and electrocardiographic artifacts are confined to much lower frequencies and do not make serious problem to accurate feature extraction.
3. SSVEP-BCI systems are relatively easy to extend to more commands.
4. Usually BCI systems have higher information transfer rates.
5. Short training phase is required and application almost does not require special training.

However, SSVEP-BCI may have also some disadvantages.

1. The flickering visual stimuli may cause some fatigue or tiredness if subjects use it for a long time. This fatigue is caused from the stimulation, while other BCI systems as P300 can cause fatigue due to the required concentration, while SSVEP does not.
2. The flickering stimuli at some frequencies, patterns, colors, and so forth may not be appropriate for subjects with photosensitive neurological disorders
3. SSVEP-based BCIs depend on muscular control of gaze direction for their operation, whereas other kinds of BCI systems do not depend on the brain's normal output pathways of peripheral nerves and muscles. Due to this reason, this paradigm may not work for some seriously disable patients. In other words, evoked potentials, especially SSVEP, require stable control of the eye muscles so that such an approach may not be applicable to all users.

In this paper, we present a BCI platform based on the SSVEP paradigm. Although the SSVEP paradigm has been exploited in a number of studies [4, 6–18, 20], our design of experiments and signal preprocessing and classification tools are innovative, moreover they are suitable for real-time applications with visual neurofeedback.

## 2. BCI SYSTEM BASED ON SSVEP PARADIGM: DESIGN AND IMPLEMENTATION

### 2.1. Stimulator design

**Figure 1:**
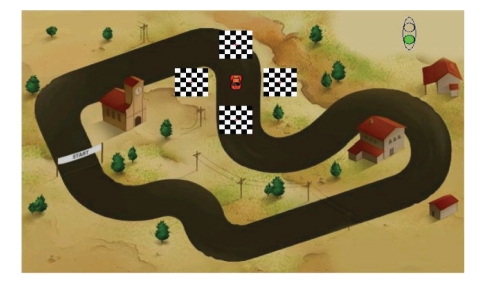
Four small checkerboards flickering at different but fixed frequencies move along with a navigated car. The subject is able to control the direction of movement of the car by focusing her/his attention on a specific checkerboard. Two sets of flickering frequencies were used: (i) low-frequency range {UP: 5 H_z_, LEFT: 6 H_z_, DOWN: 7 H_z_, RIGHT: 8 H_z_}, and (ii) medium-frequency range {UP: 12 H_z_, LEFT: 13.3 H_z_, DOWN: 15 H_z_, RIGHT: 17 H_z_}.

In this paper, we present a new BCI system with a visual stimulation unit designed as a smart multiple choice table in the form of an array of four small checkerboard images flickering with different frequencies and moving along with the controlled object (see Figure 1). When a BCI user focuses his/her attention or gazes on a specific flickering image, a corresponding periodic component (SSVEP) can be observed in the EEG signals notably over the occipital (visual) cortex [19].

When a BCI user focuses his/her attention or gaze on a specific flickering image, its corresponding weak quasi-periodic component (SSVEP) is elicited mainly over the occipital (visual) cortex [19]. In addition, they are buried in a large noise, and therefore it is a challenge to extract them reliably in real time. For this purpose, we developed and applied multistage online (real-time) signal processing tools described in detail below.

### 2.2. Analysis system overview

**Figure 2:**
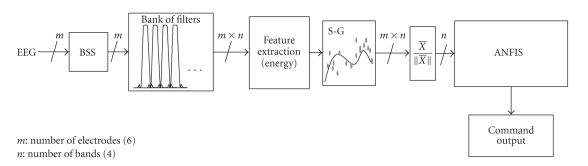
Conceptual scheme of the proposed real-time BCI system. The system consists of a BSS (blind source separation) module for automatic rejection of artifacts and noise, a bank of (narrow band-pass) filters to enhance the first harmonics of the SSVEP responses, a Feature Extraction block with S-G (Sawitzky-Golay) smoothing and energy normalization, and ANFIS (adaptive network fuzzy inference system) for a final classification.

The signal analysis unit of our BCI system consists (see Figure 2) of a data acquisition module, an enhanced signal preprocessing unit including online blind source separation (BSS) for artifact rejection and noise reduction, a bank of narrow band-pass filters, a multiple-feature extraction system with Savitzky-Golay (S-G) smoothing, energy normalization and an adaptive-network fuzzy inference system (ANFIS) [21].

To perform all signal processing tasks in real time, the analysis unit was implemented in LabVIEW and C/C++, while the stimulation unit was based on speed-optimized matlab code.

**Figure 3:**
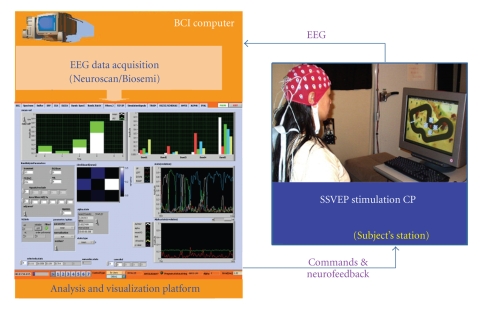
Our BCI platform consists of two PC computers. One for EEG data acquisition, stimuli generation, and a second machine for online processing of data in microbatch mode.

A general platform overview of our BCI system is shown in Figure 3.

The system is currently able to use EEG input both from the Biosemi (active-electrodes) and from the Neuroscan commercial EEG devices and is fully adaptive, accounting for the well-known large intersubject variability in the brain responses. We used only six EEG channels sampled at 256 Hz. After a very short training, two modes of operation were possible: experimental assessment mode using comparison of command requests and responses in which the success rate and the transfer rates were determined, and a free-roaming mode for overall command and control estimation. By applying BSS and a bank of subband filters, we showed that is possible to decompose and discriminate in real time at least four SSVEP waveforms with very high reliability.

**Figure 4:**
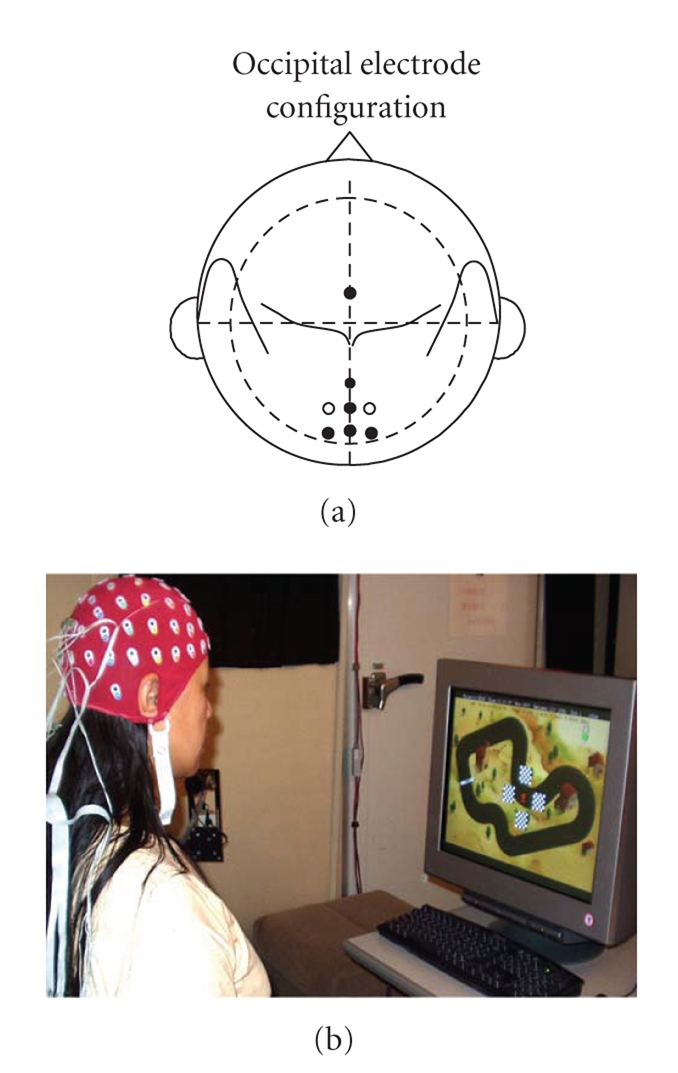
Electrode configuration. Five electrodes placed over the occipital area {CPZ, PZ, POZ, P1, P2} and one over the frontal cortex {FZ}.

In this study, we applied a set of five electrodes placed over the occipital area {CPZ, PZ, POZ, P1, P2} and one electrode placed over the frontal cortex {FZ}, as illustrated in Figure 4 (left).

### 2.3. Artifact rejection by blind source separation

A second-order blind source separation (BSS) algorithm was applied to enhance the signal and to attenuate artifacts [22]. It was characterized by a continuous working system in microbatch mode with sliding time window of four seconds and with a discrete time shifts of 120 milliseconds. This means that the system was able to refresh the incoming data every 120 milliseconds and to take into account the EEG signals from the last 4 seconds. The presence of artifacts, especially eye movement-related artifacts, can decrease the performance of the system substantially. In the case of SSVEP stimulation and analysis, their very specific response frequencies (corresponding to the observed pattern flicker frequencies) could be erroneously detected in the presence of artifacts if online BSS is not applied.

For the BSS procedure, we applied a modified and improved real-time AMUSE algorithm with time sliding windows, since such an algorithm allows a very fast (few milliseconds) and reliable estimate of the independent components with automatic ranking (sorting) according to their increasing frequency contents and/or decreased linear predictability. The implemented BSS-AMUSE algorithm can be considered as consisting of two consecutive PCA (principal component analysis) blocks. First, PCA is applied to the input data; and then a second PCA (SVD) is applied to the time-delayed covariance matrix (in our case, the delay is set to one sample or four milliseconds) of the output from the previous stage. In the first step standard or robust prewhitening (sphering) is applied as a linear transformation [22]

(1) z(t)=Qx(t),

where $Q=R_{x}^{-1/2}$ of the standard covariance matrix

(2) $$R_{x}=E\{x(t)x^{T}(t)\}$$

and x(*t*) is a vector of observed data for time instant *t*. Next, SVD is applied to a time-delayed covariance matrix of prewhitened data:

(3) $$R_{z}=E\{z(t)z^{T}(t-1)\}=USV^{T},$$

where S is a diagonal matrix with decreasing singular values and U, V are matrices of eigenvectors. Then, an unmixing (separating) matrix is estimated as

(4) W=A^−1=UTQ.

The estimated independent components are obtained as

(5) Y=WX,

where X = [x(1), x(2),…,x*(N)*.

**Figure 5:**
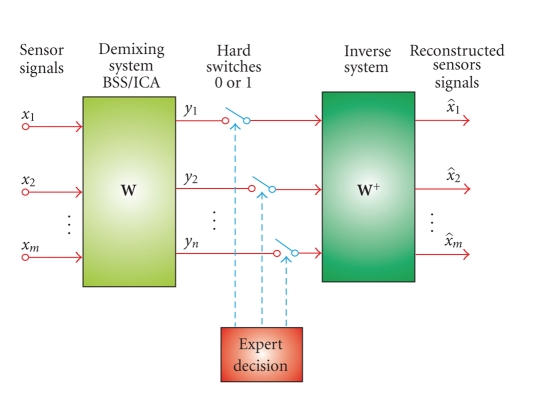
Enhancement of EEG via BSS. First, the raw EEG data (sensor signals) is decomposed and ranked as independent or spatially decorrelated components; in the next step, only the useful components are projected back to the scalp level, while undesirable components containing artifacts and noise are removed from the signal. The main advantage of our approach is that we do not need any expert decision to select significant components, since the AMUSE algorithm automatically ranks the components as illustrated in Figure 6.

The AMUSE BSS algorithm allowed us to automatically rank the EEG components. The undesired components corresponding to artifacts were removed and the rest of the useful (significant) components were projected back to scalp level using the pseudo inverse of W, see Figure 5

(6) $$\hat{X}=W^{+}X.$$

The six EEG channels were high-pass-filtered with a cutoff frequency of 2 Hz before the AMUSE algorithm was applied.

The rejection of the first and the last components had two implications: (1) the EEG signal was enhanced as some oscillations were removed which were due to ocular and other artifacts but included frequencies similar to the target flicker responses. Without this procedure, the performance of the system would have deteriorated substantially since blinking could not be avoided by the experimental subjects; (2) at the same time, we ensured that the control of the car in our BCI system was strictly due to the SSVEP responses elicited by the cortex, and not simply due to eye movements.

### 2.4. Bank of band-pass filters and features extractions

We designed a bank of third-order elliptic IIR (infinite impulse response) filters with bandwidth 0.5 Hz and with center frequencies corresponding to the flickering frequencies of the checkerboards. The fundamental frequencies of the SSVEP responses were detected by estimating the power of the output signals of the filters. We should mention here that using another type of filters could also be appropriate under the assumption that the overlap of the bandwidths of the subbands would be small enough. As we were interested only in the power of signals, their phase had no relevance in this case.

Four-time series representing the fluctuations of the energies over time were obtained and subsequently smoothed by means of a Savitzky-Golay(S-G) filter [23].

Instead of smoothing each time series' power contents in each subband with a standard moving average (MA) filter, we propose using a Savitzky-Golay filter with a second-order polynomial smoothing. The main advantage of this approach is that it tends to preserve fundamental features such as relative maxima, minima, and width of the peaks, which are usually distorted by other filtering methods, like MA. The S-G smoother approximates the time series within the moving average window not by a constant (estimate of which is the average, as in MA), but by a polynomial of higher order. In other words, this method essentially performs a local polynomial regression (of degree *M* = 2) on a distribution, of at least *k* = *n* *R* + *n* *L* + 1 points, to determine the smoothed value for each point.

The general mathematical expression of the Savitzky-Golay smoothing filter can be described as follows:

(7) $$y[n]=\overset{nR}{\underset{k=-nL}{\sum }}c_{n}x[n+k],\;$$

(8) $$c_{n}=\overset{M}{\underset{m=0}{\sum }}{\left[(A^{T}A{)}^{-1}\right]}_{0,m}n^{m},$$

where

(9) $$A_{ij}=i^{j},\;\;i=-nL,\ldots ,nR,j=0,\ldots ,M.$$

The signal is smoothed by *nL* points before, and by *nR* points after each considered time point—according to (7), where the weighting coefficients *c_n_* are obtained by means of (8). If the filter is casual, then *nR* = 0. We set *nR* > 0 to enhance the smoothing, although it introduced a small delay. For online purposes, *nR* ≪ *nL*. A moving average filter MA is a S-G filter with *M* = 0.

**Figure 7:**
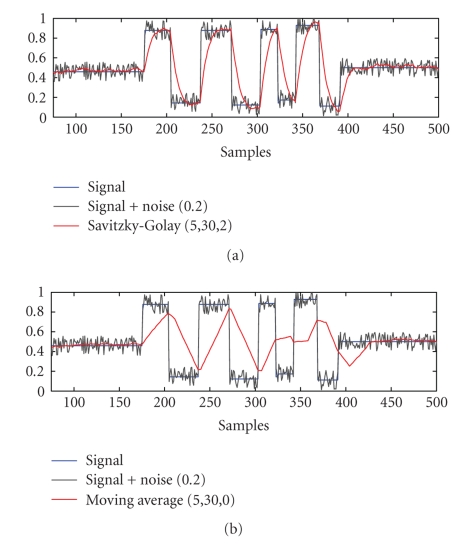
Simulated data was used in this example to show a comparison of (a) moving average smoothing (*nR* = 30, *nL* = 5) versus (b) an S-G filter (*nR* = 30, *nL* = 5, order 2). MA is not able to track short time changes having high time response. S-G moving average has similar no-noise cancellation but better track of changes. In BCI, it is important to find a good balance between enhanced smoothing and, at the same time, to be able to follow fast changes in the signal.

In Figure 7, it is shown as an example that the performance of the S-G filter is compared with a moving average filter for a simulated signal with added noise.

The S-G was applied separately for each band-pass filter and electrode.

After S-G filtering, we performed also a standard normalization of the smoothed energy as follows:

(10) $$E_{j}=\frac{\overset{M}{\underset{i=1}{\sum }}e_{ij}}{\overset{N}{\underset{j=1}{\sum }}\overset{M}{\underset{i=1}{\sum }}e_{ij}},\;\;i=1\cdots M,\;j=1\cdots N,$$

where *M* is the number of the electrodes, *N* is the number of the band-pass filters, and *e_ij_* is the estimated energy of electrode *i* and band-pass filter *j*,

(11) $$\sum_{j=1}^{M}E_{j}=1.$$

As the stimulation frequencies are close to each other, there is no need of compensation for each frequency. In case of using more frequencies, it is better to send to the classifier normalized values, although this is not the case in this paper.

**Figure 8:**
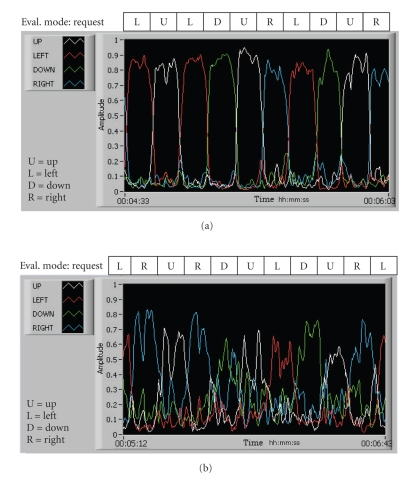
Normalized multiband signals Ej during evaluation mode: (a) a good example case with one of the subjects, and (b) a suboptimal example in another subject, where ANFIS was essential in enhancing the final performance of the system.

Therefore, *E_J_* was the relative energy per band and these energy values were used as input parameters for the ANFIS classifier, see Figure 8.

### 2.5. ANFIS classifier

One of the most complicated problems with the BCI systems is the classification of very noisy EEG signals. For this purpose, we have applied an adaptive, subject-specific classifier to recognize different SSVEPs.

The standard adaptive network based fuzzy inference system (ANFIS) architecture network was used. This system consists of a fuzzy inference system (FIS) whose membership function parameters are tuned (adjusted) using a back propagation algorithm alone in combination with a least-squares type of method (Jang, 1993) [21]. Using a hybrid learning procedure, the ANFIS can learn an input-output mapping based on some a priori knowledge (in the form of if-then fuzzy rules).

The applied ANFIS has four inputs consisted in a Sugeno-type FIS with two membership functions (generalized bell function) per input and output as a constant membership function [21]

(12) $$f(x|a,b,c)=\frac{1}{{\left(1+|x-c|/a\right)}^{2b}}.$$

Four features of EEG signals were used as input patterns (normalized energy values) for the ANFIS system, corresponding to each checkerboard.

## 3. OPERATINGMODES

To overcome the problem of the intersubject variability, some short-term preparatory activities were necessary for the BCI system before the final real-time practical evaluations or applications could be initiated. For this purpose, our BCI system was implemented to work in three separate modes.

- Training mode.
- Evaluation (testing) mode.
- Free racing (unsupervised) mode.

The training—and if necessary the evaluation modes, allowed us to find the optimal parameters for each specific subject. In this way, these parameters could be used later in the free racing (unsupervised) mode.

### 3.1. Training mode

In order to train the classifier, the computer requested the subject to fix their attention on each checkerboard {UP, LEFT, RIGHT, LEFT} during time intervals of six-seconds duration each, using voice-message requests. These requests to execute specific directions were presented in random order.

A fifth, additional, request required no stimulus and involved removing all checkerboard patterns from the screen during the six-seconds interval to measure the control non-SSVEP responses.

The corresponding values of the normalized energies were extracted for each command in the time interval between three and six seconds after each command request. In this time interval, it was considered that the subject was reaching a stable steady state for each corresponding event.

During the training mode, the neurofeedback was disconnected and the car was fixed in the center of the screen to facilitate the subject to focus her/his attention to each flickering checkerboard.

### 3.2. Evaluation mode

After the training, we asked the subject first to move the car as their own in order to confirm that he or she had the full ability to control the car in any direction. Then, to evaluate the BCI performance for this subject, including time responses and percentage of success (see results bellow), the computer generated in random order requests for movement in each direction using voice messages, similarly to the training mode. The subject was asked to move the car in one of the four directions at intervals of nine seconds in 32 trials (eight trials per direction). It was assumed that the subject successfully performed a task if she/he moved the car properly in a time window between one second and up to a maximum of six seconds after the onset of the voice-request command. During the evaluation mode, the neurofeedback was fully enabled and the car was able to move freely, responding to the subject's commands.

### 3.3. Free race (unsupervised) mode

In this mode, the user could move the car freely within the racing course (Figure 1), and we asked all the subjects to complete at least one lap to evaluate their overall control of the car by performing this task without any external voice commands. This typically took from each subject between 90 to 150 seconds to achieve this complex goal, also depending on the flicker frequency range.

## 4. EXPERIMENTAL SETTING AND RESULTS

We tested our SSVEP-based BCI system with five subjects (two females and three males) and for two different ranges of flicker frequencies: low-frequency (LF) range—5, 6, 7, 8 Hz and medium-frequency (MF) range—12, 13.3, 15, 17 Hz.

The subjects sat on a chair approximately 90 cm from the center of a 21-inch cathode-ray tube (CRT) monitor screen using a refresh rate of 120 Hz.

Six electrodes were used: five placed over the occipital cortex {CPZ, PZ, POZ, P1, P2} and one over the frontal cortex {Fz}, see Figure 2.

The performance of the BCI system was measured during the evaluation mode, as described in the previous section.

The results are shown in Table 1 (subject-specific results) and Table 2 (mean results). The data obtained in this study indicated that the performance for the medium-frequency range flicker was slightly higher when compared to the low-frequency range flicker responses, in terms of controllability of the car and execution-time delay.

Only one of the subjects was more comfortable with, and felt that his car control was better when using the low-frequency range flicker.

The subjects performed the BCI experiments just a single time for each frequency range (LF, MF), including classifier training and evaluation (results) modes. After the experiment, each subject was asked to demonstrate her/his overall control of the car for each frequency range by completing a full lap as quickly as possible in free racing mode.

## 5. CONCLUSION AND DISCUSSIONS

Although the SSVEP paradigm is well known in the BCI community since the studies performed by several research groups [6–18, 20], especially Shangkai Gao group at Tshinghua University [8–10, 18] and NASA research group of Trejo [7], we believe that our system offers several novel points for improved usability and efficiency, such as the integrated moving checkerboard patterns to maximize selective attention and to minimize eye movements in respect to the controlled target, as well as an online BSS module to reduce automatically artifacts and noise, improved feature selection algorithm with efficient smoothing and filtering and an adaptive fuzzy neural network classifier ANFIS. All of our EEG signal processing modules and algorithms are carefully optimized to work online in real time. This proposed method and BCI platform could be easily extended for various BCI paradigms, as well as for other types of brain analysis in which real-time processing and dynamic visualization of features are crucial.

Paradigms based on steady-state visual and other evoked potentials are among the most reliable modes of communication for implementation of a fast noninvasive EEG-BCI system that can discriminate in near real time a very high number of unique commands or symbols. The capability of a BCI system to issue more commands in a more reliable way has significant advantages such as allowing better control of semiautonomous remote navigation devices in hazardous environments, or navigating precisely a cursor on a computer screen (or the realization of a virtual joystick). However, in our experimental design, we have incorporated a number of original elements and ideas as compared to the typical SSVEP paradigm. In addition to our new dynamic visual stimulation approach, we have developed and implemented novel and efficient real-time signal preprocessing tools and feature extraction algorithms. Although using our dynamic pattern movement design may require some eye movement control by the subjects, as well as sustained short-term attention, the control of the object (car) could be easily changed to static for completely disabled subjects. According to our tests and to previous reports Müeller and Hillyard [24] and Kelly [9], eye movement could be avoided altogether in SSVEP (possibly at some performance cost) so that selective attention (with a fixed gaze between the flicker patterns) could be used for flicker response gating/enhancement corresponding to the requested commands.

The ability of our SSVEP-BCI system to operate not only in the medium-frequency range flicker, but also in the low-frequency range, shows its advantages in comparison to the traditionally used FFT-based methods, which usually require the usage of the higher harmonics when the visual stimulation is in the low-frequency range. In contrast, our algorithm estimates the normalized energy of each flickering frequency directly by using a dedicated tuned filter, allowing us to discriminate easily between a stimulation-driven frequency and its higher harmonics. In multiple-command BCI experimental designs, the flickering pattern frequencies could be set to be very close and limited by the minimal overlapping band-pass filters of the applied filters under the physiological constraints of discerning between cortical responses to two close stimulation frequencies.

In summary, we successfully demonstrated the application of a fast online BSS algorithm for automatic rejection of artifacts and noise reduction, a bank of band-pass filters with nonstationary smoothing, and an adaptive fuzzy classifier.

## Figures and Tables

**Figure 6:**
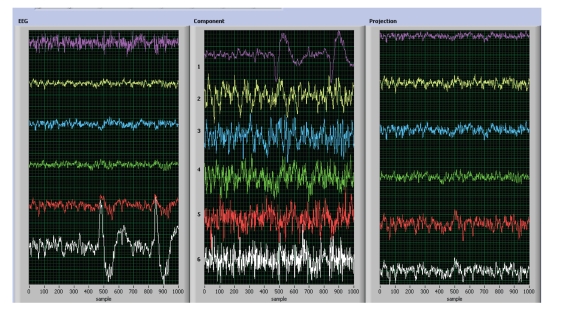
Illustration of the online preprocessing module—artifact rejection: actual EEG data (left), estimated automatically ranked independent components—the first and the last components were rejected as artifacts (center), back-projected (enhanced) EEG signals (right) which serve as the input for the bank of band-pass filters. (Four seconds window.)

**Table 1:** Experimental results for occipital configuration (mean values).

Subject	#1	#2	#3	#4	#5
	LF (5–8 Hz)				

Success (%)	100	77.5	94.8	92.3	100
Delay Time [s]	3.6 ± 0.4	3.8 ± 1.7	3.3 ± 1	3.3 ± 1.1	4.8 ± 1

	MF (12–17 Hz)				

Success (%)	100	100	100	100	82.3
Delay Time [s]	3.6 ± 0.3	3.9 ± 0.8	3.2 ± 0.4	3.1 ± 1.1	3.7 ± 1.3

**Table 2:** Experimental results for occipital configuration (mean values and mean bit rate).

Flicker range	LF	MF
(Frequency)	(5–8 Hz)	(12–17 Hz)
Success rate	93%	96.5%
Execution delay	3.7 ± 1.0 s	3.5 ± 0.8 s
Bit rate	26 bits/min	30 bits/min
